# The Apple Autophagy-Related Gene *MdATG9* Confers Tolerance to Low Nitrogen in Transgenic Apple Callus

**DOI:** 10.3389/fpls.2020.00423

**Published:** 2020-04-15

**Authors:** Liuqing Huo, Zijian Guo, Zhijun Zhang, Xin Jia, Yiming Sun, Xun Sun, Ping Wang, Xiaoqing Gong, Fengwang Ma

**Affiliations:** State Key Laboratory of Crop Stress Biology for Arid Areas/Shaanxi Key Laboratory of Apple, College of Horticulture, Northwest A&F University, Yangling, China

**Keywords:** autophagy, *MdATG9*, nitrogen starvation, apple callus, amino acid, sugar

## Abstract

Autophagy is an efficient degradation system for maintaining cellular homeostasis when plants are under environmental stress. ATG9 is the only integral membrane protein within the core ATG machinery that provides a membrane source for autophagosome formation. In this study, we isolated an *ATG9* homologs gene in apple, *MdATG9*, from *Malus domestica*. The analysis of its sequence, subcellular localization, promoter cis-elements, and expression patterns revealed the potential function of *MdATG9* in response to abiotic stressors. Overexpression of *MdATG9* in apple callus conferred enhanced tolerance to nitrogen depletion stress. During the treatment, other important *MdATG*s were expressed at higher levels in transgenic callus than in the wild type. Furthermore, more free amino acids and increased sucrose levels were found in *MdATG9*-overexpression apple callus compared with the wild type in response to nitrogen starvation, and the expression levels of *MdNRT1.1*, *MdNRT2.5*, *MdNIA1*, and *MdNIA2* were all increased higher in transgenic lines. These data suggest that, as an important autophagy gene, *MdATG9* plays an important role in the maintenance of amino acids and sugars in response to nutrient starvation in apple.

## Introduction

Plants frequently encounter various types of harsh environmental factors, such as water shortages, ion toxicity, carbon deficiency, or nitrogen depletion in the soil. To survive under these adverse conditions, plants have evolved intricate regulatory mechanisms to protect themselves against potential damage. Autophagy is one of the key biological processes for maintaining cellular homeostasis when plants are subjected to stress, which entails the degradation of superfluous or damaged organelles and aberrant proteins for recycling in cells (Li and Vierstra, 2012). Three different types of autophagy, such as micro-autophagy, macro-autophagy, and selective autophagy, have been identified in plants (Bassham et al., 2006; Marshall and Vierstra, 2018). In all three molecular routes, cytoplasmic constituents are eventually transported to the vacuole for degradation (van Doorn and Papini, 2013). Here, we focused on macro-autophagy (hereafter termed autophagy), which has been widely studied in many organisms.

Autophagy is initiated with the formation of cup-shaped phagophores elongating into a closed double-membrane vesicle called an autophagosome, which then sequesters cargo delivered to the vacuole for degradation and recycling (Bassham, 2009; Noda and Inagaki, 2015). The autophagy process relies on a dedicated set of autophagy-related (ATG) proteins which were originally identified in yeast and are largely conserved in all eukaryotes (Matsuura et al., 1997; Kang et al., 2018). These ATGs can be subdivided into several major functional clusters, including the ATG1 kinase complex, the autophagy-specific phosphatidylinositol 3-kinase complex, the Atg9 recycling system, and the Atg12 and Atg8 ubiquitin-like conjugation systems (Mizushima et al., 2011; Kim et al., 2012). ATG9, which represents the third class of ATG proteins, is the only integral membrane protein within the core ATG machinery. It has six central membrane-spanning segments with two cytosolically oriented termini involved in interactions with other ATG components (Young et al., 2006). ATG9-mediated delivery between the endomembrane and the phagophore assembly site provides an essential membrane source for vesicle nucleation (Lai et al., 2019; Zhuang et al., 2017).

Similar to autophagy studies in other organisms, isolating and identifying *ATG* genes in plants helps us to understand their molecular mechanisms. Genes for most core ATG proteins have been analyzed in plants, and various *Arabidopsis atg*-mutants, such as *atg2*, *atg4a/4b*, *atg5*, *atg7*, *atg9*, *atg10*, and *atg18a*, have been isolated to research their phenotypes (Phillips et al., 2008; Han et al., 2011). One of the major physiological roles of autophagy in plants is protecting plant cells from nitrogen depletion or carbon starvation. Several *Arabidopsis atg*-mutants are hypersensitive to nutrient-limiting (low nitrate) conditions by displaying earlier leaf senescence and lower rosette biomass (Guiboileau et al., 2013). In contrast, emerging evidence indicates that autophagy can be highly induced by abiotic or biotic stressors, and act as a central biological process involved in the plant defense system. For example, *atg5* and *atg7* mutants have been used to confirm the importance role of autophagy in heat and drought tolerance (Zhou et al., 2013).

In addition to *Arabidopsis*, the *ATG* genes in other plant species have been isolated and analyzed. *OsATG10b* has been demonstrated to play an important role in the survival of rice cells under oxidative stress (Shin et al., 2009). *ATG5-* or *ATG7-*silenced tomato mutants display compromised heat tolerance compared with the wild type (Zhou et al., 2013). *SiATG8a* from foxtail millet plays a vital role conferring tolerance to both nitrogen starvation and drought stress (Li et al., 2015). The maize autophagy mutant *atg12* has been used to reveal the specific aspects of maize metabolism that are controlled by autophagy (McLoughlin et al., 2018). In our previous study, we characterized *MdATG18a* in apple, and demonstrated that it enhances plant resistance to drought stress (Sun et al., 2018a), nitrogen depletion (Sun et al., 2018b), and heat stress (Huo et al., 2020a).

The autophagic recycling process contributes to remobilize nutrients under a nutrient deficient condition, providing amino acids, fatty acids, and sugars for plant survival (Signorelli et al., 2019). In response to a nutrient limitation, autophagy-mediated amino acid metabolism seems to play an important role in plant resistance. For example, in cases of limited carbohydrates, amino acids produced from protein degradation are an important source of alternative substrates for energy supply, and autophagy has a potential role in energetic maintenance under this condition (Avin-Wittenberg et al., 2015; Barros et al., 2017). Moreover, decreased levels of free amino acids in *Arabidopsis* seedlings, particularly in *atg* mutants, have been reported following carbon limitation (Avin-Wittenberg et al., 2015). These results exemplify the essential role of autophagy for maintaining free amino acid levels during starvation conditions. In addition to amino acid metabolism, the modulation of carbon metabolism is also important for plant cells to survive under starvation conditions. In our previous study, leaves of *MdATG18a*-overexpressing apple plants accumulated more soluble sugars compared with the wild type under a nitrogen-depletion treatment (Sun et al., 2018b).

In both mammals and yeast, *ATG9* deficiency inhibits the formation of autophagosomes, particularly *atg9*-knockout mice present an embryonic lethal condition (Saitoh et al., 2009; Yamamoto et al., 2012). Consistent with the essential role of *ATG9* in mammals and yeast, depleting *ATG9* in plants leads to an extensive accumulation of abnormal autophagosome-related tubular structures when autophagy is induced, indicating the essential and specific role of *ATG9* in the autophagic degradation pathway (Zhuang et al., 2017). Here, we isolated an *ATG9* homologs gene, *MdATG9*, in apple. After analyzing its sequence, subcellular localization, and promoter cis-elements as well as its expression patterns in apple plants under abiotic stress, we revealed that, as an important autophagy gene, *MdATG9* might possess the conserved function in response to abiotic stressors. Overexpression of *MdATG9* in apple callus enhanced their tolerance to a nitrogen starvation treatment. Furthermore, the levels of free amino acids were less reduced and sucrose content increased more in *MdATG9*-overexpressing apple callus compared with the wild type under low-nitrogen stress.

## Materials and Methods

### Plant Materials, Growing Conditions, and Treatments

Mature leaves were collected from three 2 years old apple (*Malus domestica* Borkh. “Golden Delicious”) plants growing at the Horticultural Experimental Station of Northwest A&F University (Yangling, Shaanxi, China) for cloning *MdATG9* and its promoter.

Seeds of *M. hupenensis* were stratified at 4°C for 60 days, and the seedlings were grown in pots for 3 months in a greenhouse (25°C, 14 h photoperiod). The response of seedlings to H_2_O_2_, methyl viologen (MV), or exogenous abscisic acid (ABA) were examined by spraying the leaves with 10 mM H_2_O_2_, 50 μM MV, or 100 μM ABA, respectively. The response of seedlings to salinity was examined by irrigating the plants to saturation with 200 mM NaCl. Seedlings were exposed to 4 or 42°C to test their responses to low or high temperature, respectively. For each assay mentioned above, leave samples were collected at 0, 2, 4, 8, 12, and 24 h. Drought stress was induced by withholding irrigation from plants that had previously been fully watered. Seedlings for the nitrogen-starvation assay were transferred to a chamber for hydroponic culture. After a 15 days preincubation, Ca(NO_3_)_2_ and KNO_3_ in Hoagland’s nutrient solution were replaced with CaCl_2_ and KCl, respectively. In both cases, leaves were sampled on days 0, 2, 4, 6, 8, and 10. In all treatment types mentioned above, three biological replicates were prepared with five seedlings combined as one replicate. All collected samples were frozen rapidly in liquid nitrogen and stored at −80°C.

Seedlings of wild-type *Arabidopsis thaliana* L. (Columbia) and two homozygous T_3_ transgenic lines were used. They were sterilized and germinated on a standard Murashige and Skoog (MS) medium and cultured under a 14 h photoperiod at 25°C. Five days old uniformly sized seedlings grown on MS agar plates were transferred vertically into unmodified media or nitrogen-limited media (nitrogen concentration reduced from 60 to 10 mM) for the nitrogen-starvation and osmotic stress assays. Fresh weights and root lengths were measured from 5 plates on day 15 after transfer.

Wild type and transgenic “Orin” apple calluses were used, based on treatments described previously (Wang et al., 2017a). Briefly, 0.1 g portions of 15 days old similar and well-grown calluses of wild type and transgenic lines were transferred to MS control media or low-nitrogen media with the nitrogen concentration lowered from 60to 5 mM. Fresh weights were measured from 10 plates on day 15 after transfer. Frozen samples were collected from 15 plates and stored at −80°C.

### Sequence Analysis, Construction of Plasmids, and Subcellular Localization

Homologous sequences and putative conserved domains were predicted from the NCBI BLASTp programs. The sequences were aligned with homologs sequences from other species using DNAMAN. Phylogenetic trees were constructed with MEGA 5.0 software (Tamura et al., 2011), using the neighbor-joining method with 1,000 bootstrap replicates. Putative cis-regulatory elements in the *MdATG9* promoter region were examined with the PlantCARE program.

To construct the vectors for subcellular localization and for the transgenic lines, the *MdATG9* coding region was introduced into the pGWB405-GFP and pCambia2300 vectors, both driven by the CaMV 35S promoter and carried by the kanamycin (Kan) selectable marker in plants. The sequencing-confirmed plasmids were transformed into *Agrobacterium tumefaciens* strain EHA105 by electroporation. The primers used for constructing the vector are listed in Supplementary Table S2.

For subcellular localization, leaves of 5 weeks old tobacco (*Nicotiana tabacum*) were transiently transformed as described previously (Yang et al., 2000). Three days later, green fluorescent protein (GFP) signals in transformed tobacco leaves were observed using confocal microscopy, and the images were processed with FV10-ASW software.

### Transformation of *Arabidopsis* and Apple Callus

The “Columbia” *Arabidopsis* ecotype was transformed using the floral dip method with *Agrobacterium* contained *MdATG9*-pCambia2300 vector (Zhang et al., 2006). Transgenic seeds (T_1_) were selected on MS medium supplemented with 50 mg L^–1^ Kan. After confirmation by polymerase chain reaction (PCR), T_2_ homozygous lines were obtained after selecting transgenic lines at a 3:1 segregation ratio, and the T_3_ seeds were collected.

Calluses of “Orin” apple were used for apple callus transformation. The calluses were cultured on solid MS medium containing 1.0 mg L^–1^ 2,4-D, 1.0 mg L^–1^ 6-BA, and 8 g L^–1^ agar in the dark at 25°C. The apple calluses were transformed referring to a method described previously (Wang et al., 2016, 2018). Briefly, 7 days old suspension callus grown in a liquid medium were mixed with *Agrobacterium* contained *MdATG9*-pCambia2300 vector and rotated gently for 10 min at 25°C for transformation. After 2 days co-cultivation on solid medium, the callus was washed three times with sterile water containing 400 mg L^–1^ cefotaxime, and then transferred to a selection medium supplemented with 400 mg L^–1^ cefotaxime and 30 mg L^–1^ kanamycin for transgene selection. After 3–5 serial subcultures, resistant calluses showing stable growth were subjected to PCR and qRT-PCR for analysis of the transgene.

### RNA Extraction, DNA Isolation, and qRT-PCR Analysis

Total RNA was extracted using the Wolact plant RNA isolation kit (Wolact, Hong Kong, China), and first-strand cDNA was synthesized using the RevertAid First Strand cDNA Synthesis Kit (Thermo Fisher Scientific, Waltham, MA, United States) with 1 μg total RNA. Genomic DNA was isolated with the Wolact Plant Genomic DNA purification kit. qRT-PCR was performed according to the SYBR Premix Ex Taq II Kit (Takara, Dalian, China) on a LightCycler 96 (Roche, Basel, Switzerland). Three biological replicates were tested in each assay, and representative data from one repetition were shown. Values were calculated using *malate dehydrogenase* (*MDH*) as the endogenous control (Perini et al., 2014). The relative expression level of each gene was calculated according to the 2^–ΔΔ*CT*^ method (Livak and Schmittgen, 2001), and a dissociation curve analysis was performed to determine the specificity of each gene. The gene-specific primers are shown in Supplementary Table S2.

### Measurements of Soluble Sugars and Amino Acids

Soluble sugars were extracted and derivatized sequentially with methoxamine hydrochloride and N-methyl-N-trimethylsilyl-trifluoroacetamide, as described previously (Hu et al., 2018). The metabolites were analyzed with a Shimadzu GCMS-2010 SE (Shimadzu Corp., Kyoto, Japan). Values were calculated based on their corresponding standard curves and internal standards.

Amino acids were extracted and measured as described previously (Huo et al., 2020b). Briefly, 200 mg of frozen leaf samples were extracted in 2 ml 50% ethanol (including 0.1 M HCl) and centrifuged at 13,000 g for 10 min. The supernatant was added to methanol at a final volume of 10 ml. The samples were filtered through a 0.22 μm filter to analyze the metabolites with a liquid chromatography-mass spectrometry system (QTRAP5500; SCIEX, Concord, ONT, Canada) equipped with an Inertsil ODS-4 C18 column (4.6 × 250 mm, 5 μm) at a flow rate of 0.3 ml/min. The solvent system consisted of water containing 0.1% (v/v) formic acid (A) and acetonitrile (B). Data were quantified by comparing the peak surface areas with those obtained using standard amino acids (Sigma-Aldrich, St. Louis, MO, United States).

### Statistical Analysis

SPSS 16.0 software (SPSS Inc., Chicago, IL, United States) was used for the statistical analysis. Three independent replicates were used for each determination. Experimental data are presented as mean ± standard error. The statistical analysis was performed by one-way analysis of variance followed by Tukey’s multiple range test. A *p* < 0.05 was considered significant.

## Results

### Molecular Cloning, Sequence Analysis, and Subcellular Localization of *MdATG9*

**FIGURE 1:**
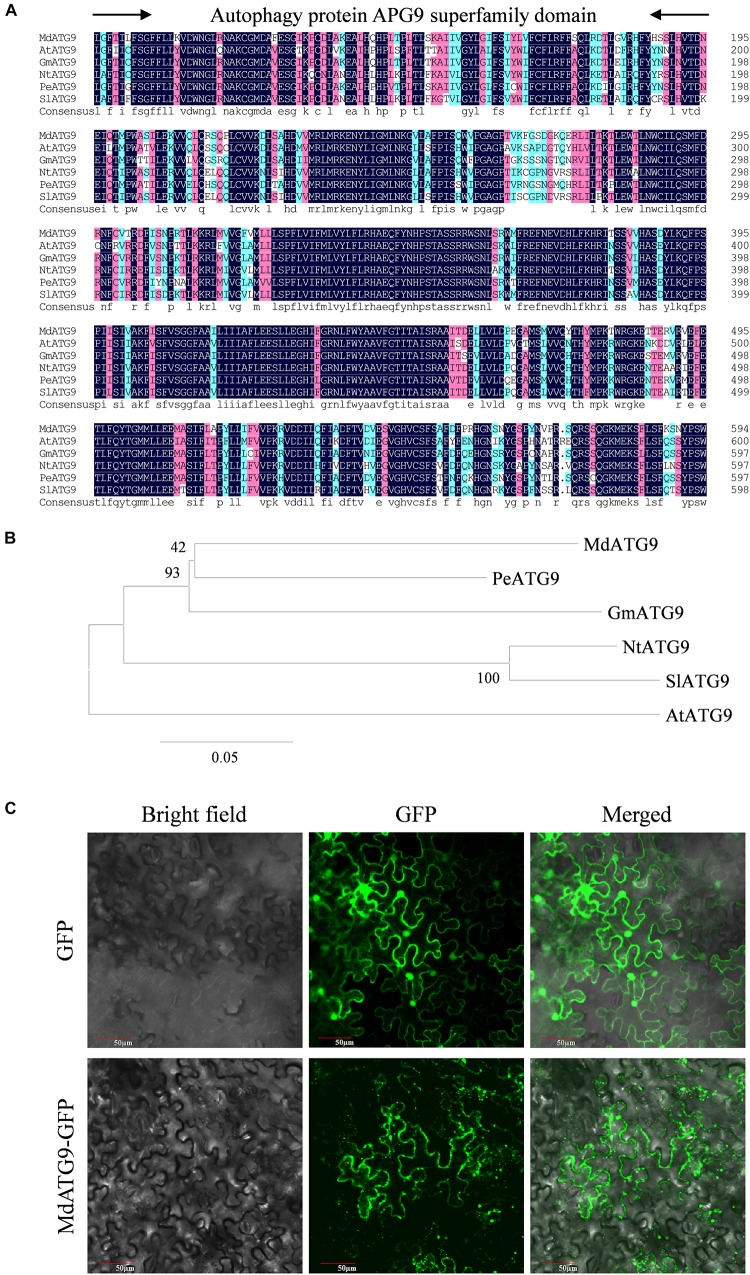
Sequence analysis and cellular localization of MdATG9. **(A)** Alignment of deduced ATG9 amino acid sequence from *Malus domestica* (*Md*), *Arabidopsis thaliana* (*At*), *Populus euphratica* (*Pe*), *Glycine max* (*Gm*), *Nicotiana tabacum* (*Nt*), and *Solanum lycopersicum* (*Sl*). **(B)** Phylogenetic analysis of MdATG9 protein with ATG9 proteins from other species. **(C)** Subcellular localization analysis of MdATG9-GFP fusion protein in tobacco epidermal cells.

We identified a homologous sequence of *AtATG9* from *M. domestica* through homologous cloning and named it *MdATG9*. The gene contained a 2,622 bp open reading frame (ORF) and encoded an 874 amino acid-deduced protein. Protein alignment revealed high homology between the MdATG9 protein and other ATG9 proteins from *A. thaliana* (64%), *Glycine max* (70%), *Nicotiana tabacum* (67%), *Populus euphratica* (72%), and *Solanum lycopersicum* (66%) (Figure 1A). All of these aligned sequences had the conserved autophagy protein APG9 superfamily domain. The phylogenetic tree analysis indicated that the MdATG9 protein formed a close cluster with PeATG9 and GmATG9 (Figure 1B).

To examine the subcellular localization of MdATG9, we fused its ORF with the GFP at the N-terminus under control of the CaMV35S promoter. For transient expression in tobacco epidermal cells, the MdATG9–GFP fusion protein was detected in the cytoplasm, while GFP alone was distributed throughout the cell (Figure 1C).

In addition, we analyzed the promoter region of *MdATG9* using the PlantCARE database and revealed the presence of several recognized stress-responsive *cis*-elements (Supplementary Figure S1 and Supplementary Table S1).

### Expression Analysis of *MdATG9* Under Abiotic Stress

**FIGURE 2:**
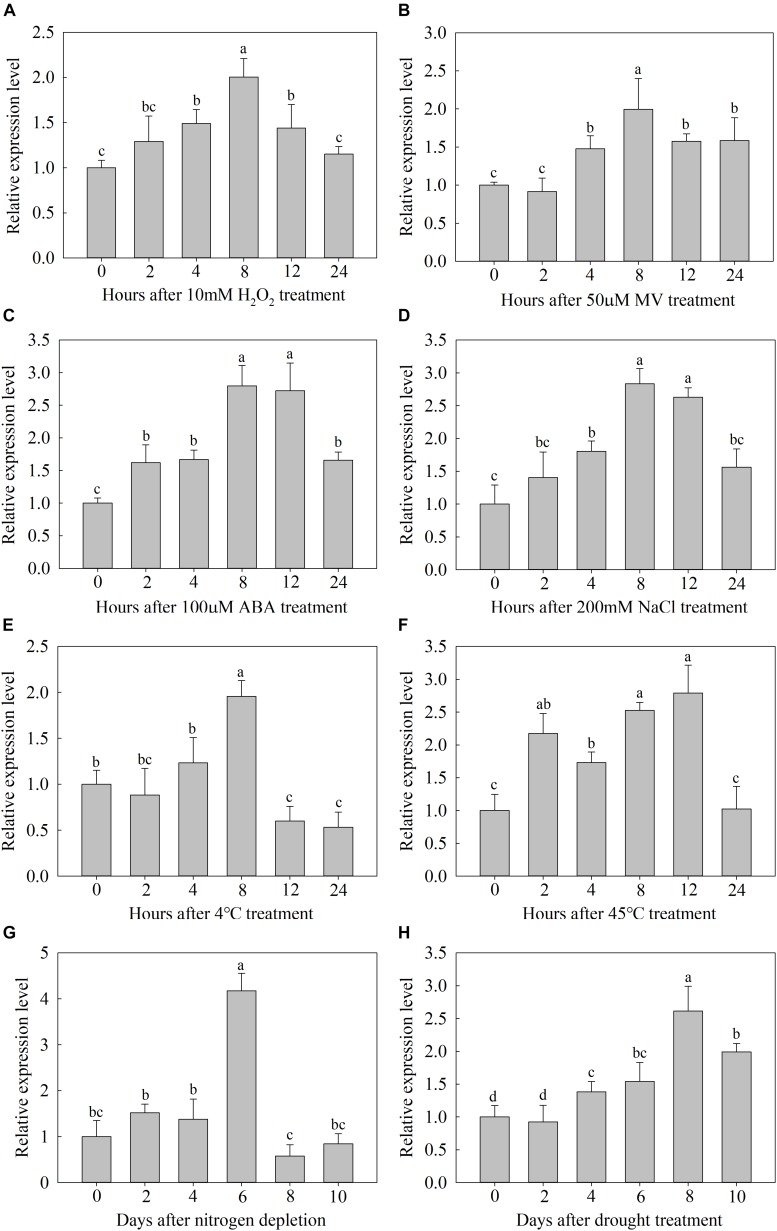
Expression analysis of *MdATG9*. Relative expression **(A)** under H_2_O_2_ treatment, **(B)** MV treatment, **(C)** ABA treatment, **(D)** NaCl stress, **(E)** low temperature, **(F)** high temperature, **(G)** nitrogen depletion treatment, and **(H)** drought stress. Total RNA was extracted from leaf samples. Data are the means of three replicates with SEs. Different letters indicate significant differences between treatments, according to one-way ANOVA and Tukey’s multiple range test (*P* < 0.05).

To investigate the possible functions of *MdATG9*, we examined it expression in the plants of *M. hupenensis* in response to several abiotic stressors. In response to H_2_O_2_ or MV, the expression of *MdATG9* increased by approximately twofold at 8 h (Figures 2A,B). ABA or NaCl treatment also induced it expression, with both transcripts were upregulated more than 2.7-fold after 8 h of exposure (Figures 2C,D). Under the low temperature treatment (4±C), expression increased by approximately twofold 8 h after treatment and then decreased below the initial level (Figure 2E). High temperature (45±C) upregulated the transcripts continually, with expression being almost two and threefold higher at 2 and 12 h, respectively (Figure 2F). In response to nitrogen starvation, *MdATG9* expression was induced approximately fourfold on day 6. In addition, the transcripts were significantly upregulated in response to drought treatment, by more than 2.5-fold on day 8 (Figure 2G). These data suggest that *MdATG9* was induced by most stress conditions, particularly by nitrogen starvation.

### Overexpression of *MdATG9* Enhances Tolerance to Nitrogen Starvation in Apple Callus

**FIGURE 3:**
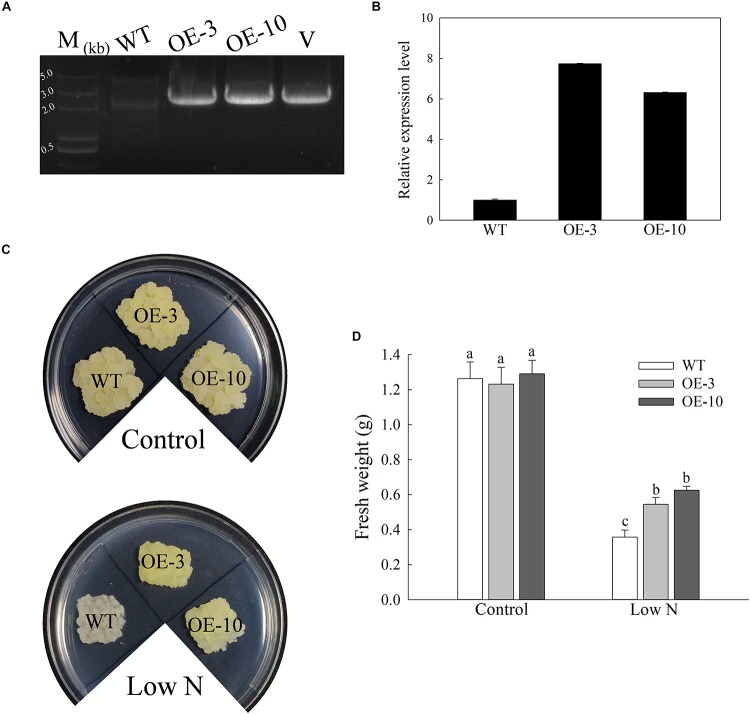
Overexpression of *MdATG9* in apple callus confers enhanced tolerance to nitrogen starvation. **(A)** PCR with gDNA. Lanes M, molecular marker DL5000; WT, non-transformed wild-type; OE-3 and OE-10, *MdATG9*-transgenic callus lines; V, positive plasmid control. **(B)** qPCR analysis of *MdATG9* transcripts in apple callus lines OE-3 and OE-10. **(C)** Phenotypes of WT and transgenic apple callus grew on normal MS media or nitrogen-limitation media for 15 days. **(D)** Fresh weights of callus after 15 days treatment. Data are the means of ten replicates with SE. Different letters indicate significant differences between treatments, according to one-way ANOVA and Tukey’s multiple range test (*P* < 0.05).

As several stress-responsive *cis*-acting elements were found in the *MdATG9* promoter region, and its transcription was induced by several abiotic stressors, we generated overexpressing (OE) “Orin” lines that over-expressed *MdATG9*, as confirmed by PCR with gDNA and qRT-PCR (Figures 3A,B). The mRNA transcripts increased by 7.7- and 6.3-fold in OE-3 and OE-10, respectively (Figure 3B).

After 15 days of growth on MS medium, the similar and well-grown calluses of the wild type and transgenic lines were transferred to MS control or low-nitrogen media (Figure 3C). The initial weight of all lines was controlled at 0.1 g, then we observed and measured their growth after 15 days of stress treatment. No significant difference was observed between the wild type (WT) and OE lines in terms of growth characteristics or fresh weights when grown on control MS medium. After 15 days of nitrogen deprivation, the WT callus had stopped growing and turned white, while the two transgenic lines grew faster and were slightly yellow (Figures 3C,D). These results show that overexpression of *MdATG9* improved the tolerance of nitrogen starvation in apple callus.

Furthermore, we obtained two transgenic *Arabidopsis* lines for the further analyses on its potential functions under low-nitrogen condition. We selected the independent T_3_ homozygous lines and vertically transferred the 5 days old *Arabidopsis* seedlings of wild type and transgenic lines into unmodified media or nitrogen-limited media (Supplementary Figures S2A–C). After 15 days of nitrogen deprivation, both the fresh weights and root lengths of the OE lines were significantly higher than those of the wild type, and the transgenic lines grew more lateral roots than the wild type under the low-nitrogen treatment (Supplementary Figures S2C–E). These results suggest that ectopic expression of *MdATG9* in *Arabidopsis* alleviated the negative effects of nitrogen starvation stress.

### Overexpression of *MdATG9* in Apple Callus Upregulates the Expression of Other *MdATG*s Under Stress Conditions

**FIGURE 4:**
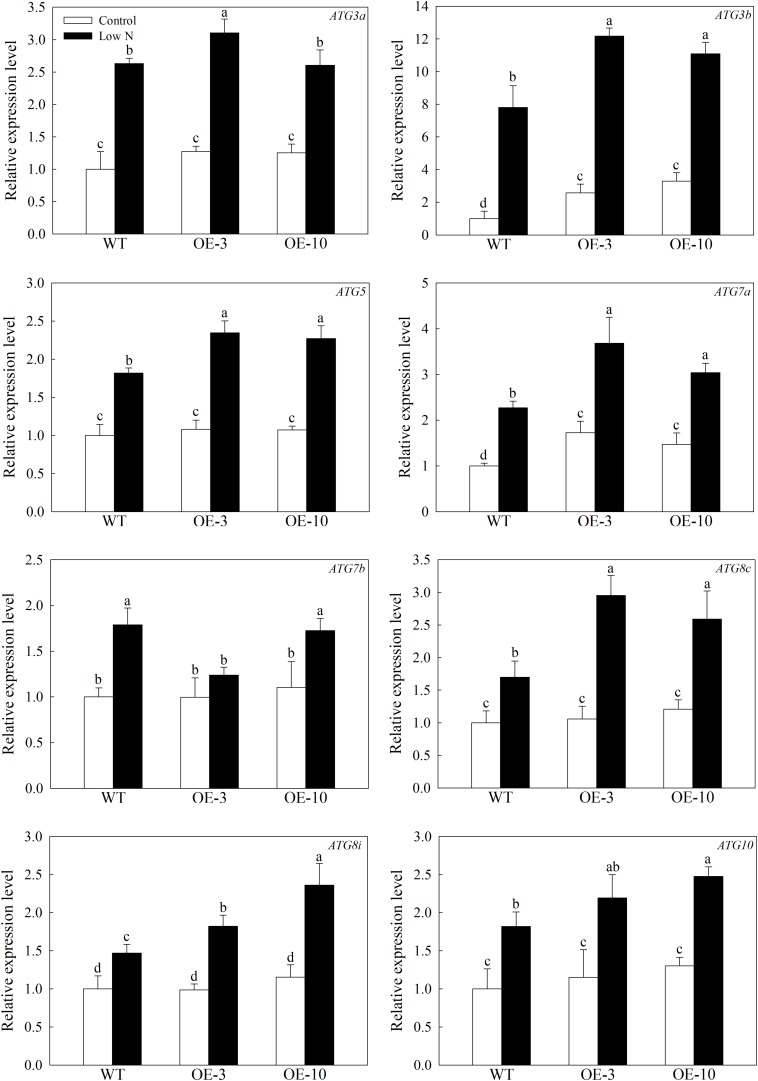
Changes in the expression of other *MdATGs* in WT and *MdATG9*-OE apple callus following nitrogen starvation. Data are the means of three replicates with SEs. Different letters indicate significant differences between treatments, according to one-way ANOVA and Tukey’s multiple range test (*P* < 0.05).

To investigate the occurrence of autophagy among genotypes under stress conditions, we used qRT-PCR to examine the expression patterns of 8 other important *MdATG*s. The expression of *MdATGs* was induced significantly by nitrogen starvation (Figure 4). Under the control condition, the expression of *MdATG3a*, *MdATG5*, *MdATG7b*, *MdATG8c*, *MdATG8i*, and *MdATG10* did not differ among genotypes, except *MdATG3b* and *MdATG7a*, were expressed at higher levels in the OE lines than in the WT. Under the low-nitrogen treatment, the *MdATG*s were all expressed at higher levels in the OE lines, except for the expression of *MdATG7b*, which was lower in OE-3 than in the WT. These results suggest that the transcripts of other important *ATG* genes were more responsive to the nitrogen-starvation condition in the OE lines than in the WT.

### Amino Acid Metabolism Is Involved in *MdATG9*-Mediated Low-Nitrogen Tolerance

**FIGURE 5:**
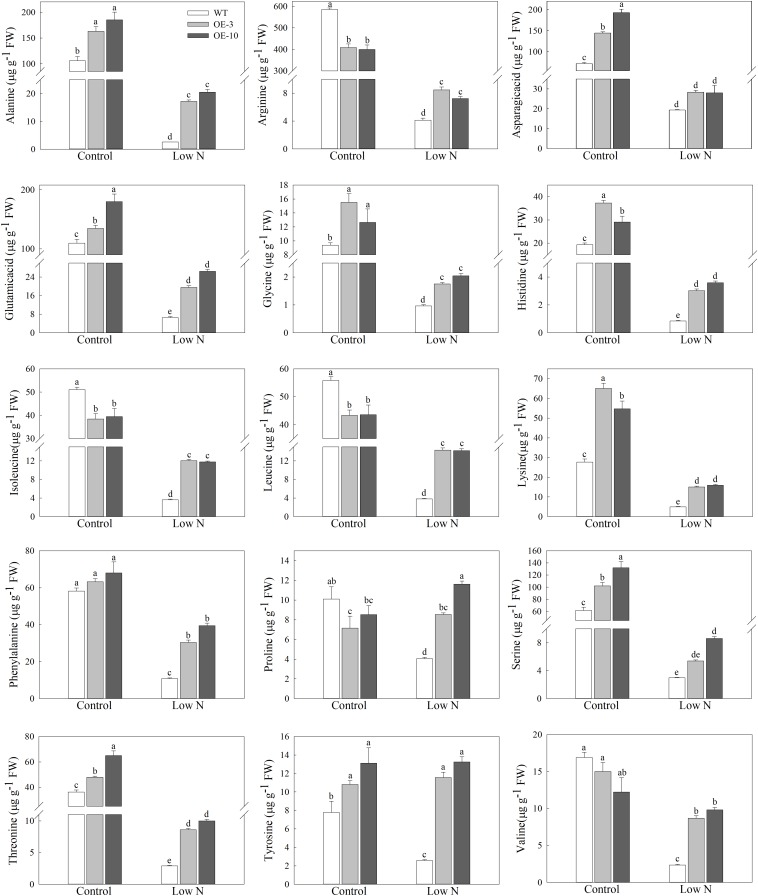
Amino acids levels in WT and *MdATG9*-OE apple callus after nitrogen starvation treatment, as measured by LC-MS. Data are the means of three replicates with SE. Different letters indicate significant differences between treatments, according to one-way ANOVA and Tukey’s multiple range test (*P* < 0.05).

The maintenance of free amino-acid levels is essential for plants under nutrient-starvation conditions (Onodera and Ohsumi, 2005). We measured the concentrations of 15 amino acids to further investigate whether *MdATG9* was involved in amino acid metabolism in response to nitrogen starvation in the apple callus (Figure 5). The low-nitrogen treatment led to a significant decrease in the levels of most amino acids in the apple callus. Several amino acids with higher content in the apple callus under control conditions, such as Ala, Arg, Glu, and Ser, were reduced 10–50 times by the nitrogen-starvation treatment. However, at the end of the treatment, the concentration of each amino acid, except Asp, was significantly higher in the OE lines than those in the WT. For example, the levels of Ala and Tyr in the transgenic lines were almost 7- and 5-fold that of the WT, respectively after treatment. In addition, we observed that Pro content was reduced by the nitrogen-starvation treatment only in the WT callus, and it increased slightly in the OE lines. These results demonstrate that overexpression of *MdATG9* alleviated the limitation of nitrogen-starvation to the size of the free amino-acid pool in the apple callus.

**FIGURE 6:**
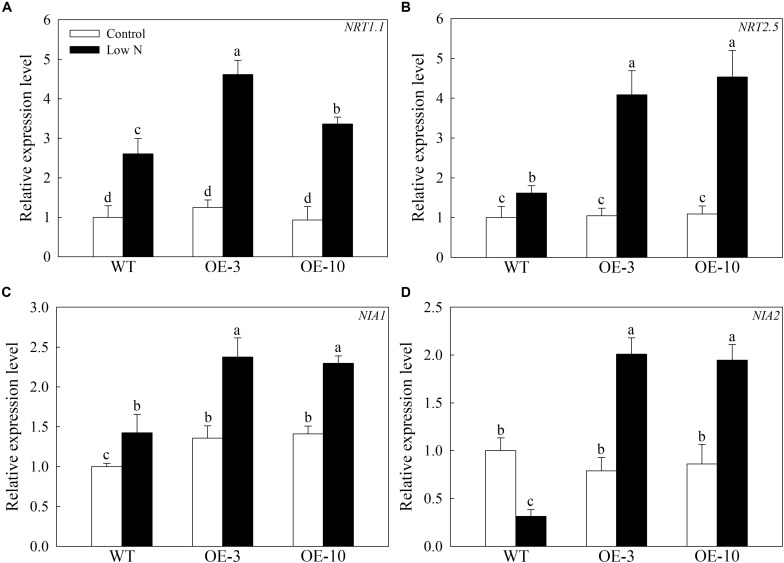
Changes in transcript levels of four genes involved in nitrate uptake following nitrogen starvation. Changes in expression of **(A)** *MdNRT1.1*, **(B)** *MdNRT2.5*, **(C)** *MdNIA1*, and **(D)** *MdNIA2* under nitrogen-starvation stress. Data are the means of three replicates with SEs. Different letters indicate significant differences between treatments, according to one-way ANOVA and Tukey’s multiple range test (*P* < 0.05).

To examine whether *MdATG9* influences N-signaling in apple, we investigated the transcript levels of several genes involved in nitrate uptake. As shown in Figure 6, the expression levels of *MdNRT1.1*, *MdNRT2.5*, and *MdNIA1* increased under the low-nitrogen treatment, and they were all expressed higher in the OE lines than in the WT. For example, the *MdNRT2.5* transcript level in the transgenic lines was almost three times that of the WT after treatment (Figure 6B). In addition, the *MdNIA2* transcripts decreased threefold in the WT callus under treatment, whereas they were upregulated in the OE lines (Figure 6D). These data demonstrate that overexpression of *MdATG9* upregulated some genes responsible for nitrate assimilation therefore increasing nitrate utilization in the apple callus.

### Sugar Metabolism Is Involved in *MdATG9*-Mediated Low-Nitrogen Tolerance

**FIGURE 7:**
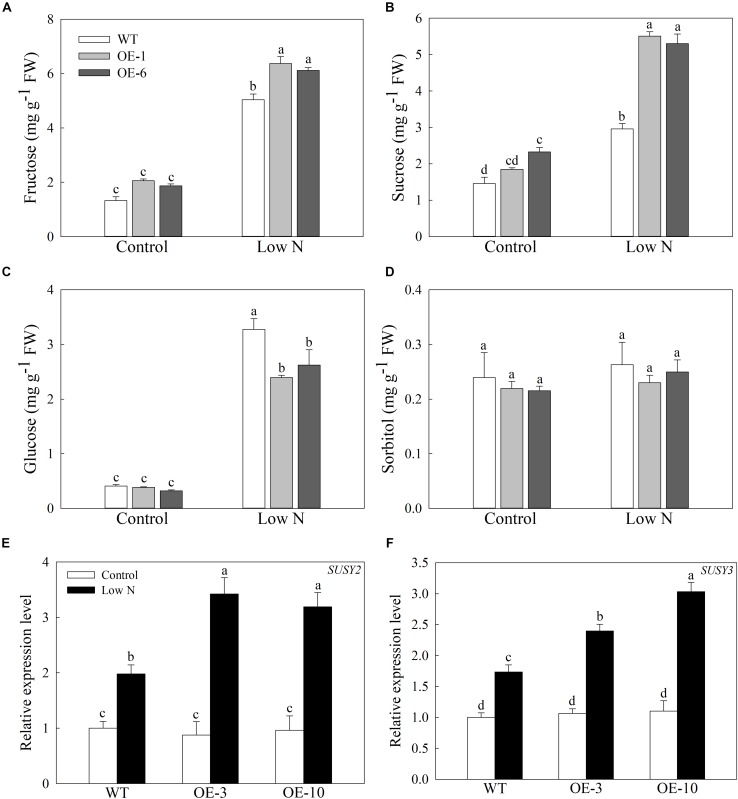
Accumulation of soluble sugars in apple callus under low-nitrogen treatment. **(A)** Fructose, **(B)** sucrose, **(C)** glucose, and **(D)** sorbitol contents in apple callus after nitrogen starvation treatment. Changes in expression of **(E)** *MdSUSY2* and **(F)** *MdSUSY3* under nitrogen-starvation stress. Data are the means of three replicates with SEs. Different letters indicate significant differences between treatments, according to one-way ANOVA and Tukey’s multiple range test (*P* < 0.05).

In response to nitrogen starvation, the carbon metabolism of plant cells is remodeled to a certain extent, so we measured the concentrations of soluble sugars in apple calluses under a low-nitrogen treatment (Sun et al., 2018b). The nitrogen starvation treatment greatly increased the contents of fructose, glucose, and sucrose in the apple callus, but the changes in sorbitol concentration are inconspicuous (Figure 7). Although the levels of fructose rose more in the OE lines than in the WT, and the concentrations of glucose were lower in the OE lines than the WT after treatment (Figures 7A,C). In addition, the levels of sucrose were slightly higher in the OE lines even under the control conditions, but were approximately 1.8-fold higher when compared with WT calluses under the deficit treatment (Figure 7D). Furthermore, the expression levels of *MdSUSY2* and *MdSUSY3*, which convert sucrose into fructose in apple, were induced by the low-nitrogen treatment and increased more in the transgenic lines than in the WT (Figures 7E,F). These data suggest that overexpression of *MdATG9* improves the accumulation of sucrose in apple calluses in response to a low-nitrogen condition, with more conversion to fructose than glucose.

## Discussion

Autophagy is an intracellular catabolic process in which cellular components are enclosed by double membrane-bounded vesicular structures called autophagosomes and transported into vacuoles (Bassham, 2009; Noda and Inagaki, 2015). As the only transmembrane protein within the core ATG machinery, ATG9 has long been suggested to provide a membrane source for autophagosomes and to interact with several other functional ATG proteins at the site of autophagosome formation (Zhuang et al., 2017; Lai et al., 2019). Here, we isolated *MdATG9* from apple, and characterized it function in response to nitrogen starvation stress in *Arabidopsis* and apple callus. The MdATG9 protein had a conserved autophagy protein APG9 superfamily domain and presented high homology with other ATG9s from various plant species (Figures 1A,B). Through a subcellular localization analysis, we found that the MdATG9–GFP fusion protein occurred in the cytoplasm of tobacco epidermal cells, with a multiple puncta appearance consistent with a previous report in yeast (Figure 1C; Noda et al., 2000). Then, we isolated the *MdATG9* promoter region and bioinformatically analyzed several stress- or hormone-responsive cis-acting elements in the promoter (Supplementary Figure S1). Consistent with this, the expression analysis under abiotic stress conditions revealed that *MdATG9* was induced by most stress conditions, particularly by nitrogen starvation (Figure 2).

For further evaluation of the function of *MdATG9* in coping with stress, we generated transgenic *Arabidopsis* plants and apple calluses that overexpressed *MdATG9*. After treatment with the nitrogen starvation stress, overexpression of *MdATG9* led to enhanced stress tolerance in both *Arabidopsis* plants and apple callus (Figure 3 and Supplementary Figure S2). Previous studies in *Arabidopsis* have reported that the *atg9* mutant shows a relatively later and less severe phenotype of senescence compared with *atg5-1* and *atg7-2* mutants during an extended dark treatment (Barros et al., 2017). Although *ATG9* is essential for the formation of autophagosomes, the *atg9* mutant displayed a milder reduction in the autophagic process, which explains the relatively minor phenotype observed in this mutant line (Shin et al., 2014; Zhuang et al., 2017). In this study, we found that most detected genes were expressed at higher levels in the transgenic lines than in the WT after examining the expression patterns of other important *MdATG*s in response to low-nitrogen treatment in all apple callus lines (Figure 4). These results indicate that overexpression of *MdATG9* in apple callus might promote the activity of other autophagy gene under nitrogen starvation stress.

In our earlier investigation, we isolated some autophagy-related genes in apple, and found that the tolerance to limited supplies of nutrition increases after these genes were over-expressed, regardless of whether *Arabidopsis* or an apple callus was used in the tests (Wang et al., 2016, 2017a,b). In this study, we not only demonstrated that overexpression of *MdATG9* increased the tolerance to a limited supply of nitrogen, but also detected higher transcript levels of other important *MdATG*s in *MdATG9*-OE apple calluses. We measured the concentrations of 15 amino acids, considering that the overexpression of *MdATG9* might participate in amino acid metabolism in response to nitrogen starvation in apple callus. Although the low-nitrogen treatment led to a significant decrease in amino acid levels, the concentrations of most amino acids were significantly higher in the transgenic lines than in the WT. Protein synthesis is dramatically limited by intracellular amino acid levels under nitrogen starvation, and autophagy appears to be essential for the size of the free amino acid pool (Onodera and Ohsumi, 2005). Here, we discovered that overexpression of *MdATG9* in apple calluses alleviated the limitation of nitrogen starvation to the free amino acid level (Figure 5); therefore, alleviating the limitation of protein synthesis, which contributed to a milder restriction on growth.

In addition, we found that although the levels of most of the detected amino acids were severely down-regulated by nitrogen starvation among genotypes, the contents of Pro and Try increased slightly in transgenic apple callus (Figure 5). Tyr is a substrate for dopamine, which was reported to promote the tolerance of apple to nutrient deficiency-induced stress (Liang et al., 2017). As a compatible solute, Pro has been reported to have antioxidant properties. Recent study reported that the role of plant autophagy in response to salt stress was associated with proline accumulation (Huo et al., 2020b). Combined with the results that the Tyr and Pro content was non-decreasing but increasing in transgenic apple callus in response to nitrogen starvation, the accumulation of both Tyr and Pro appears to contribute, at least in part to *MdATG9*-mediated tolerance to nitrogen starvation. However, the definite relationship between *MdATG9* and Tyr and Pro accumulation requires further exploration.

The high-affinity nitrate transporter NRT2 in *Arabidopsis* accounts for most of the high-affinity nitrate influx activity under a nitrogen-limited condition (Leran et al., 2014). Here, we determined that the *MdNRT2.5* transcript level was induced at higher levels by nitrogen starvation in the transgenic lines than in the WT (Figure 6). Nitrate reductase (NIA) is a key enzyme involved in nitrate assimilation after its uptake in plants (Nawaz et al., 2017). In the present study, *MdNIA2* transcripts decreased in WT calluses under treatment, whereas they were upregulated in the OE lines. These results suggest that overexpression of *MdATG9* in the callus might promote the absorption of a small amount of nitrate in low-nitrogen medium, and further promote nitrate assimilation; therefore, increasing nitrate utilization in apple callus.

In addition, as the sufficient sucrose was present in the medium, the limited amino acids in the plant cell might be re-utilized directly for protein synthesis rather than used as an energy source. As the energy source of callus growth, the concentrations of soluble sugars in apple calluses also changed in response to the low-nitrogen treatment. In our previous study, we demonstrated that *MdATG18a*-OE apple plants accumulate more soluble sugars compared with the WT under a nitrogen-depletion treatment (Sun et al., 2018b). Here, we found that fructose, glucose, and sucrose contents in apple calluses increased significantly under the low-nitrogen treatment (Figure 7). However, unlike these three sugars all accumulated higher in *MdATG18a*-OE apple plants after the deficit treatment, we found that the levels of glucose were lower in *MdATG9*-OE apple callus while the levels of sucrose in the OE lines were almost two times that of the WT in response to low nitrogen (Figures 7B,C). It might because that the sugars needed for growth of the callus are derived from absorbing the sucrose from the medium. We speculated that overexpression of *MdATG9* might improve the sucrose accumulation in apple callus by enhancing absorption of sucrose from the low-nitrogen medium, with more conversion to fructose than glucose. Moreover, considering that both free amino acids and soluble sugars are important compatible osmolytes in the cytoplasm of plant cells (Barros et al., 2017), the significant increases of soluble sugars in apple callus might be able to alleviate the osmotic pressure caused by the large decrease in free amino acids in response to nitrogen starvation.

**FIGURE 8:**
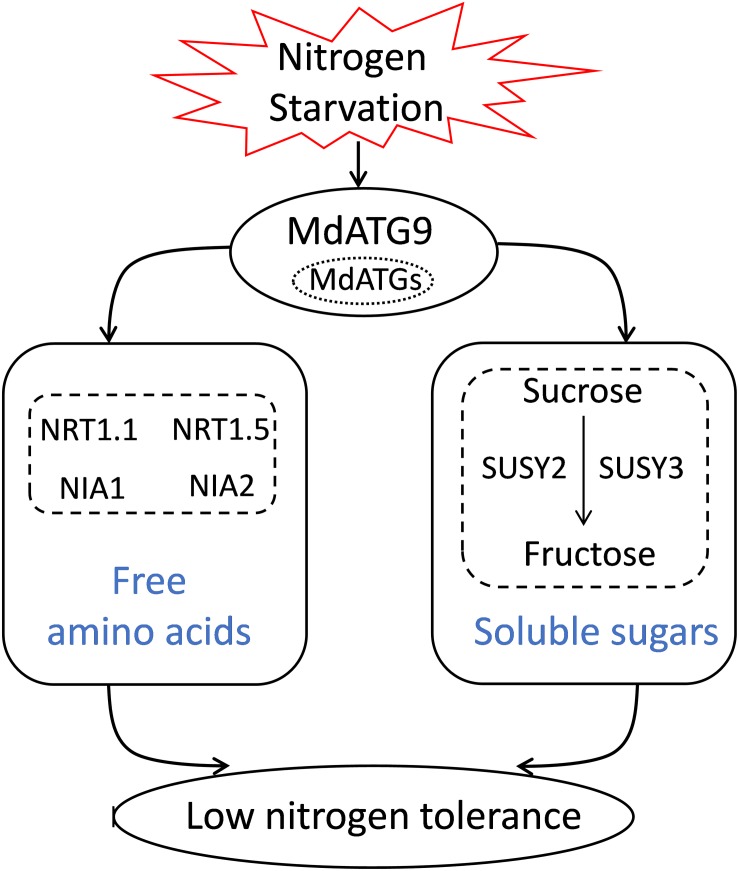
A proposed model for explaining the function of apple *MdATG9* in response to nitrogen starvation.

In summary, we isolated and characterized *MdATG9* in apple and examined its function by overexpression in *Arabidopsis* and apple calluses. The results showed that overexpression of *MdATG9* could enhance plant tolerance to nitrogen starvation stress (Figure 8). The free amino acid analysis revealed that *MdATG9* overexpression lessened the reduction of most amino acid concentrations under the low-nitrogen treatment, and promoted nitrate assimilation and utilization. In addition, *MdATG9* overexpression improved the accumulation of soluble sucrose in apple callus by enhancing the absorption of sucrose from low-nitrogen medium; therefore, resulting in better growth of the transgenic callus. Therefore, the current study characterized the function of *MdATG9* under a nitrogen starvation condition, and explored the role of *MdATG9* in response to nitrogen starvation from the perspectives of amino acid and sugar metabolism. These findings provide insight into the metabolic importance of *MdATG9* acted as an important autophagy gene in response to nutrient starvation in apple.

## Data Availability Statement

The raw data supporting the conclusions of this article will be made available by the authors, without undue reservation, to any qualified researcher.

## Author Contributions

FM, XG, and LH designed the experiments. LH, ZG, and ZZ performed the experiments and analyzed the data, assisted by XJ, YS, XS, and PW. LH, XG, and FM wrote the manuscript with contributions from all authors.

## Conflict of Interest

The authors declare that the research was conducted in the absence of any commercial or financial relationships that could be construed as a potential conflict of interest.
