# Supplementary material for: The Apple Autophagy-Related Gene MdATG9 Confers Tolerance to Low Nitrogen in Transgenic Apple Callus
Source: Front Plant Sci. 2020 Apr 15;11:423. doi: 10.3389/fpls.2020.00423 (PMC7174617; doi:10.3389/fpls.2020.00423)
Supplement: Supplementary file 1 [file Data_Sheet_1.docx]

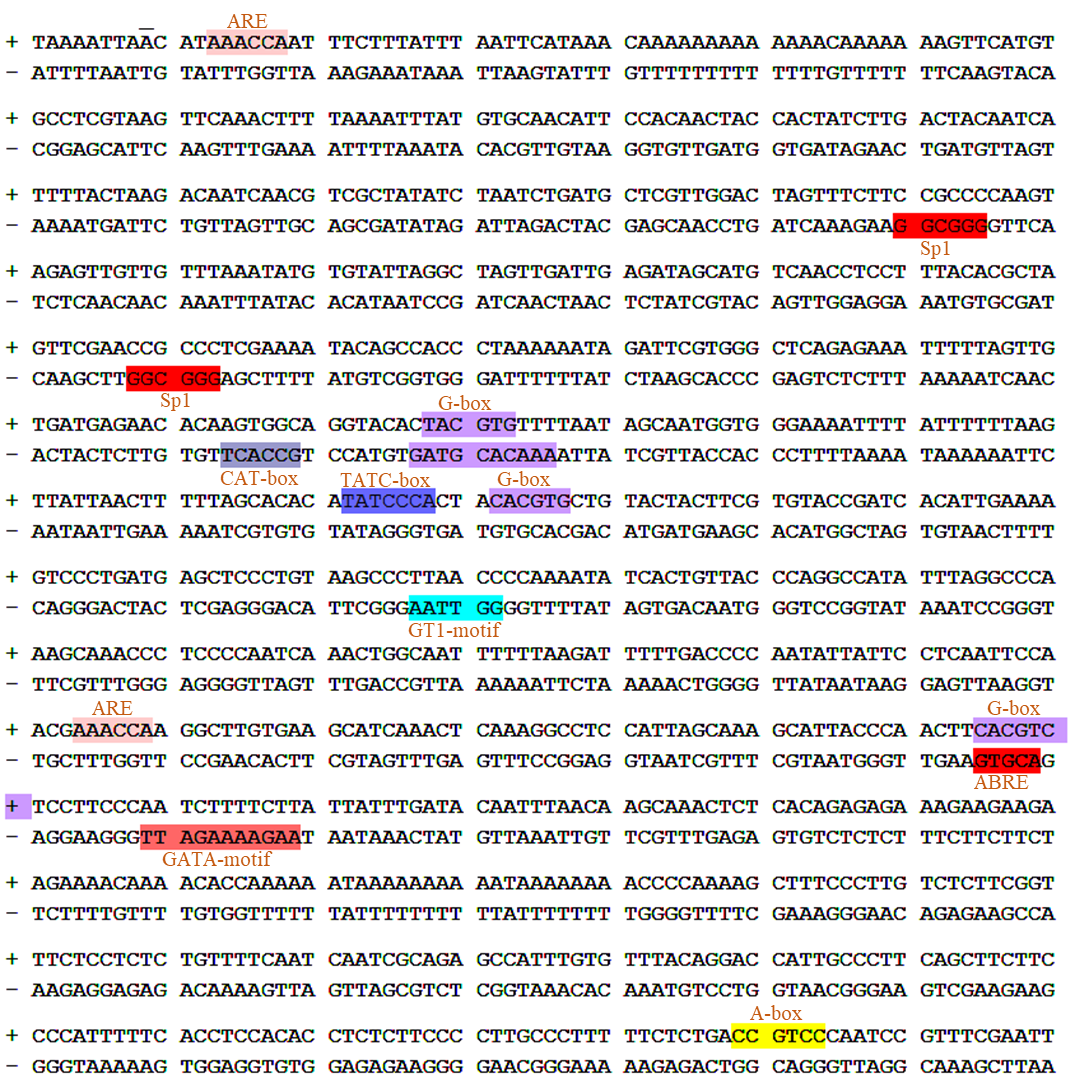

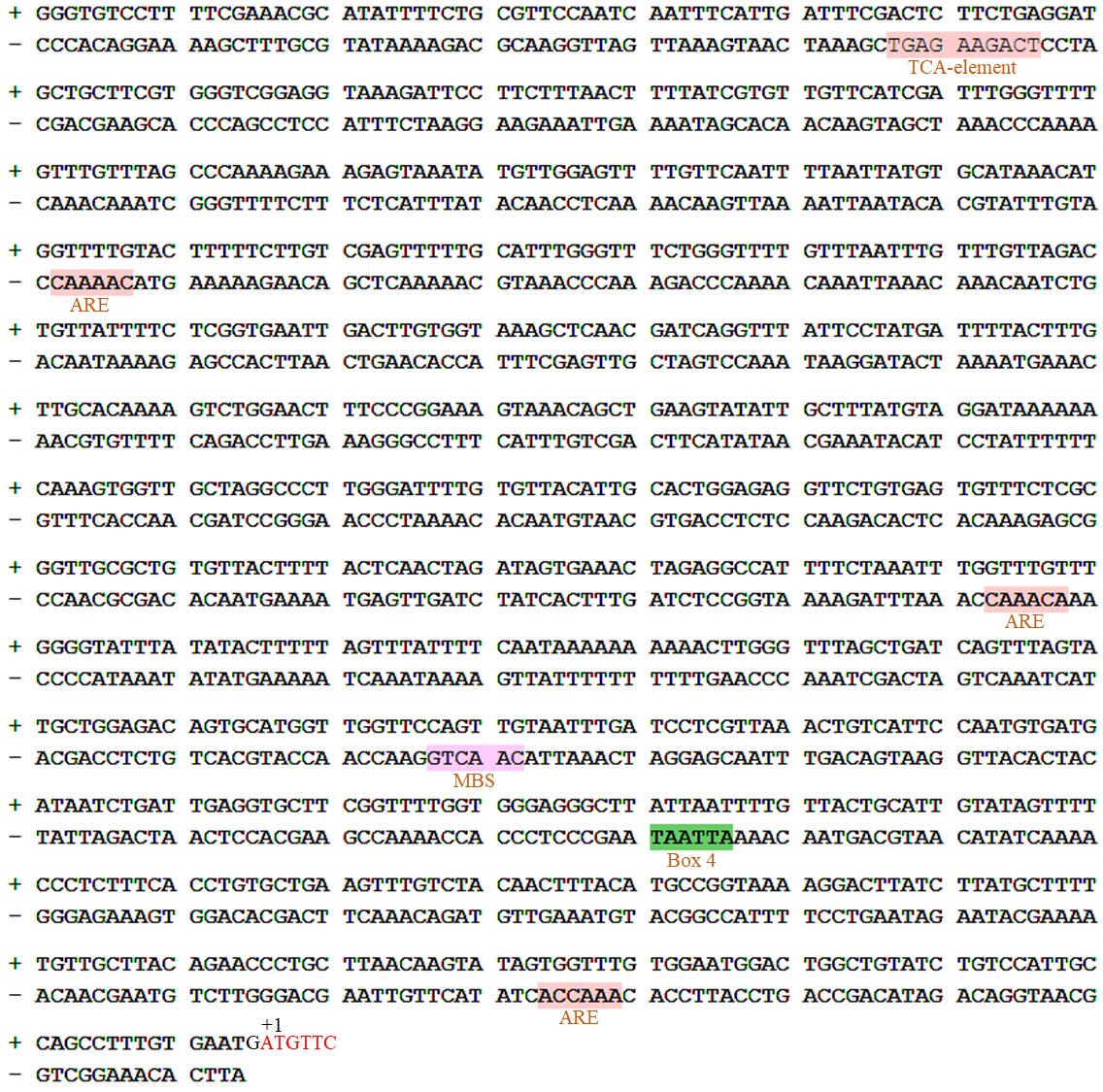


Fig. S1 Putative *cis*-regulatory elements in promoter region of *MdATG9*, as predicted from PlantCARE database. Letters in red indicates the coding sequence of *MdATG9*.


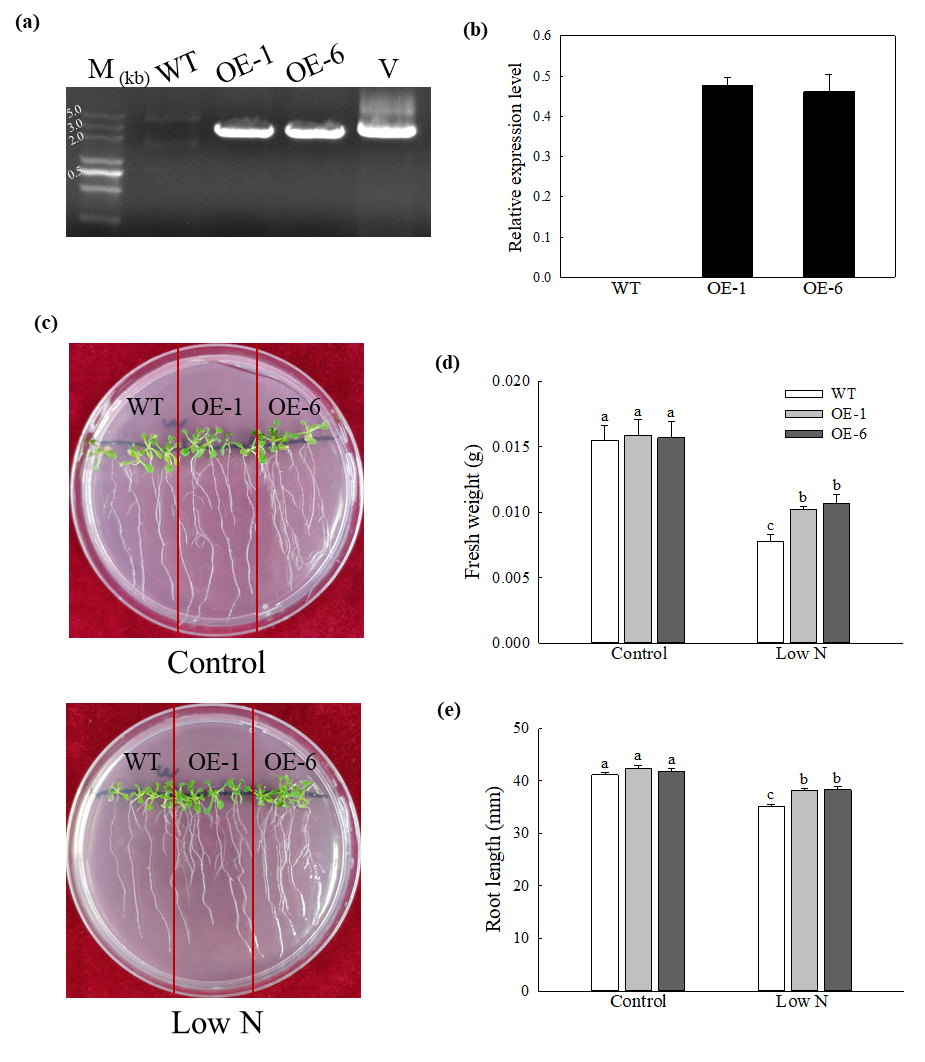


Fig. S2 Heterologous expression of *MdATG9* in *Arabidopsis* confers enhanced tolerance to nitrogen starvation. (a) PCR with gDNA. Lanes M, molecular marker DL5000; WT, non-transformed wild-type; OE-1 and OE-6, *MdATG9*-transgenic lines; V, positive plasmid control. (b) qPCR analysis of *MdATG9* transcripts in *Arabidopsis* lines OE-1 and OE-6. (c) Phenotypes of WT and transgenic *Arabidopsis* plants grew on normal MS media or nitrogen-limitation media for 15d. Plant (d) fresh weights and (e) root lengths are recorded. Data are the means of ten replicates with SE. Different letters indicate significant differences between treatments, according to one-way ANOVA and Tukey’s multiple range test (P <0.05).

Supplemental Table1 Predicted *cis*-regulatory elements with putative functions identified in promoter of *MdATG9* using PlantCARE database.

| *Cis*-element | Positions | Sequences | Putative function |
| --- | --- | --- | --- |
| ARE | 13 (+) | AAACCA | cis-acting regulatory element essential for the anaerobic induction |
|  | 634 (+) | AAACCA |  |
|  | 1190 (-) | AAACCA |  |
|  | 1531 (-) | AAACCA |  |
|  | 1854 (-) | AAACCA |  |
| Sp1 | 200 (-) | GGGCGG | light responsive element |
|  | 288 (-) | GGGCGG |  |
| G-box | 377 (-) | TAACACGTAG | cis-acting regulatory element involved in light responsiveness |
|  | 378 (+) | TACGTG |  |
|  | 452 (+) | CACGTG |  |
|  | 695 (+) | CACGTC |  |
| CAT-box | 364 (-) | GCCACT | cis-acting regulatory element related to meristem expression |
| TATC-box | 442 (+) | TATCCCA | cis-acting element involved in gibberellin-responsiveness |
| GT1-motif | 517 (-) | GGTTAA | light responsive element |
| ABRE | 695 (-) | ACGTG | cis-acting element involved in the abscisic acid responsiveness |
| GATA-motif | 709 (-) | AAGATAAGATT | part of a light responsive element |
| TCA-element | 1037 (-) | TCAGAAGAGG | cis-acting element involved in salicylic acid responsiveness |
| MBS | 1637 (-) | CAACTG | MYB binding site involved in drought-inducibility |
| Box 4 | 1721 (-) | ATTAAT | part of a conserved DNA module involved in light responsiveness |

Supplemental Table2 Primers used in this study

| **Gene** | **Sequence (5**'**-3**'**)** | **Purpose** |
| --- | --- | --- |
| *ATG9* | F: ATGTTCGGTAGGTTGAAGGGTG | cloning Sequence |
|  | R: CTATGGCTTTATTTCCGGAGTTTCT |  |
| s*ATG9* | F: GGGGACAAGTTTGTACAAAAAAGCAGGC  TTCATGTTCGGTAGGTTGAAGGG | Vector construction for sub-cellular localization |
|  | R: GGGGACCACTTTGTACAAGAAAGCTGGG  TTTGGCTTTATTTCCGGAGTTT |  |
| *ATG9pro* | F: TTCATGTGCCTCGTAAGTTCAAAC | Cloning the promoter of *MdATG9* |
|  | R: CATTCACAAAGGCTGGCAATGG |  |
| oe*ATG9* | F: CGGGATCCATGTTCGGTAGGTTGAAGGG | Vector construction for plant transformation |
|  | R: GGGGTACCTGGCTTTATTTCCGGAGTTT |  |
| d*ATG9* | F: GAGAACACGGGGGACTCTAGA | DNA confirmation of *MdATG9* for transgenic identification |
|  | R: CGATCGGGGAAATTCGAGCTC |  |
| q*ATG9* | F: GTTTATTGCAGACTTCACGG | Quantitative expression of *MdATG9* |
|  | R: GCCTCTTGGAGGAGAATATG |  |
| *MDH* | F: CGTGATTGGGTACTTGGAAC | Reference gene used in real-time PCR |
|  | R: TGGCAAGTGACTGGGAATGA |  |
| q*ATG3a* | F: AAGGGGGCGGAGATGGTTC | Quantitative expression of *MdATG3a* |
|  | R: GCACTTAGAGACGAGGTTATCGC |  |
| *qATG3b* | F: AGGGAGATGGTTTTGAAACAGA | Quantitative expression of *MdATG3b* |
|  | R: ACTTAGAGACGAGGTTATCGC |  |
| q*ATG5* | F: GCAGGTCGTGTTCCAGTTC | Quantitative expression of *MdATG5* |
|  | R: CCTCCTCCTCCTTGTATCTCAA |  |
| *qATG7a* | F: GCGGATATGAGCAACCTTGGC | Quantitative expression of *MdATG7a* |
|  | R: ATCAATAGGCGCAACGACATCA |  |
| q*ATG7b* | F: ATCGGTAACAGGAGTAAGTCGG | Quantitative expression of *MdATG7b* |
|  | R: TTTATCAAGCGCATGAAAGCCT |  |
| q*ATG8c* | F: GCGTTCAAGATGGAGCACCCTC | Quantitative expression of *MdATG8c* |
|  | R: CAGCCCTTTCCACAACCACTGG |  |
| q*ATG8i* | F: GCAGCAGGCTTCACTTGACTCC | Quantitative expression of *MdATG8i* |
|  | R: GGAATCCATGCGACTGGCTGTT |  |
| q*ATG10* | F: TGGAACCAGCGAGTGGATGAAG | Quantitative expression of *MdATG10* |
|  | R: ACAACTGAGAGCCAAGACACCA |  |
| q*NRT1.1* | F: TCCTTTATGCTCTGCTTGCTC | Quantitative expression of *Md**NRT1.1* |
|  | R: GGGACTGTGGTTGAGATGGT |  |
| q*NRT2.5* | F: TCGATCTGAAACTCCACACG | Quantitative expression of *MdNRT2.5* |
|  | R: ACTTCTTCGCCACAACATCC |  |
| q*NIA1* | F: AACCCGCCGATAAACAGAC | Quantitative expression of *MdNIA1* |
|  | R: GTTCGCAGTTGAAGGGATGT |  |
| q*NIA2* | F: CGGGAAGAAAGTCACACGAG | Quantitative expression of *MdNIA2* |
|  | R: CCAGAAACACCAGCACCAGT |  |
| q*SUSY2* | F: TGTGGTTGGTGGTTACATGGATG | Quantitative expression of *MdSUSY2* |
|  | R: GCTGCTATCCATCGGAACTGAC |  |
| q*SUSY3* | F: TTATGGTTTCTGGAAGTATGTGTC | Quantitative expression of *MdSUSY3* |
|  | R: GTCGATGGCTTCAGGAACAGATT |  |
